# *catena*-Poly[copper(I)-di-μ-bromido-copper(I)-bis­[μ-4-methyl-1*H*-1,2,4-triazole-5(4*H*)-thione-κ^2^ *S*:*S*]]

**DOI:** 10.1107/S1600536812014444

**Published:** 2012-04-13

**Authors:** Saowanit Saithong, Jonathan Charmant, Chaveng Pakawatchai

**Affiliations:** a Department of Chemistry and Center for Innovation in Chemistry, Faculty of Science, Prince of Songkla University, Hat Yai, Songkhla 90112, Thailand; b School of Chemistry, University of Bristol, Bristol BS8 1TS, England

## Abstract

In the title coordination polymer, [CuBr(C_3_H_5_N_3_S)]_*n*_, the Cu^I^ atom adopts a tetra­hdral CuS_2_Br_2_ coordination geometry arising from two *S*-bonded 4-methyl-1*H*-1,2,4-triazole-3(4*H*)-thione ligands and two bromide ions. Both the S and Br atoms act as bridging ligands, connecting pairs of Cu^I^ atoms and generating chains propagating in [100]. Inter-chain N—H⋯N hydrogen bonds generate layers in the *ac* plane. Weak intra-chain N—H⋯Br inter­actions also occur.

## Related literature

For related structures of metals coordinated by 1,2,4-triazole derivatives, see: Cingi *et al.* (1996 ▶); Haasnoot (2000 ▶); Kajdan *et al.* (2000 ▶); Menzies & Squattrito (2001 ▶); Klingele & Brooker (2003 ▶). 
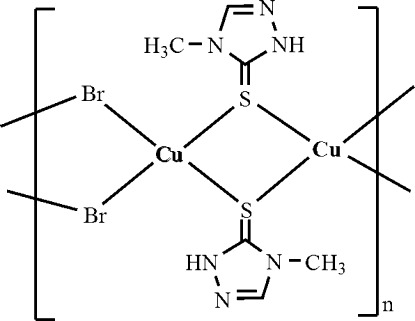


## Experimental

### 

#### Crystal data

[CuBr(C_3_H_5_N_3_S)]
*M* *_r_* = 258.62
Monoclinic, 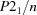

*a* = 5.5781 (11) Å
*b* = 12.931 (3) Å
*c* = 9.810 (2) Å
β = 97.69 (3)°
*V* = 701.2 (2) Å^3^
*Z* = 4
Mo *K*α radiation
μ = 9.02 mm^−1^
*T* = 100 K
0.28 × 0.12 × 0.06 mm

#### Data collection

Bruker D8 CCD diffractometer
Absorption correction: multi-scan (*SADABS*; Bruker, 1998) *T*_min_ = 0.284, *T*_max_ = 0.582
7830 measured reflections
1610 independent reflections
1513 reflections with *I* > 2σ(*I*)
*R*_int_ = 0.043

#### Refinement

*R*[*F* ^2^ > 2σ(*F* ^2^)] = 0.027
*wR*(*F* ^2^) = 0.056
*S* = 1.16
1610 reflections
86 parameters
1 restraint
H atoms treated by a mixture of independent and constrained refinement
Δρ_max_ = 0.64 e Å^−3^
Δρ_min_ = −0.37 e Å^−3^

### 

Data collection: *SMART* (Bruker, 2003) ▶; cell refinement: *SAINT* (Bruker, 2003) ▶; data reduction: *SAINT* and *SHELXTL* (Sheldrick, 2008 ▶); program(s) used to solve structure: *SHELXS97* (Sheldrick, 2008 ▶); program(s) used to refine structure: *SHELXL97* (Sheldrick, 2008 ▶); molecular graphics: *Mercury* (Macrae *et al.*, 2008) ▶; software used to prepare material for publication: *Mercury* (Macrae *et al.*, 2008) ▶ and *publCIF* (Westrip, 2010 ▶).

## Supplementary Material

Crystal structure: contains datablock(s) I, global. DOI: 10.1107/S1600536812014444/hb6718sup1.cif

Structure factors: contains datablock(s) I. DOI: 10.1107/S1600536812014444/hb6718Isup2.hkl

Additional supplementary materials:  crystallographic information; 3D view; checkCIF report

## Figures and Tables

**Table:** 

Cu1—S1	2.3124 (9)
Cu1—S1^i^	2.4012 (9)
Cu1—Br1	2.4638 (7)
Cu1—Br1^ii^	2.5085 (8)

**Table:** 

Cu1—Br1—Cu1^ii^	67.81 (2)
Cu1—S1—Cu1^i^	73.60 (3)

Symmetry codes: (i) 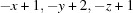; (ii) 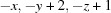.

**Table 2:** Hydrogen-bond geometry (Å, °)

*D*—H⋯*A*	*D*—H	H⋯*A*	*D*⋯*A*	*D*—H⋯*A*
N1—H1⋯N2^iii^	0.86 (2)	2.35 (3)	2.890 (4)	121 (3)
N1—H1⋯Br1	0.86 (2)	2.78 (2)	3.566 (3)	153 (3)

Symmetry code: (iii) 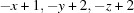.

**Fig. 1.**
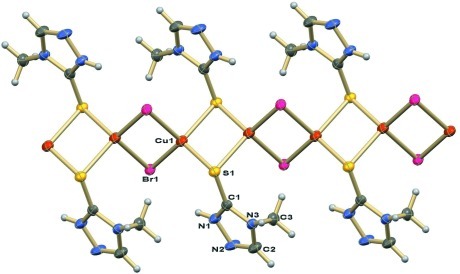
A view of the 1-D polymeric chain of [Cu(µ2-Hmptrz)(µ2-Br)]n with displacement ellipsoids plotted at the 50% probability level.

**Fig. 2.**
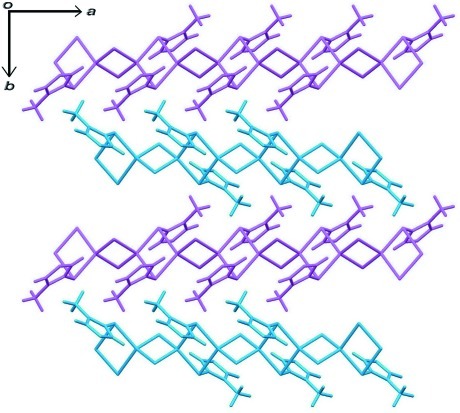
The arrangement of the polymeric chains in packing of [Cu(µ2-Hmptrz)(µ2-Br)]n.

**Fig. 3.**
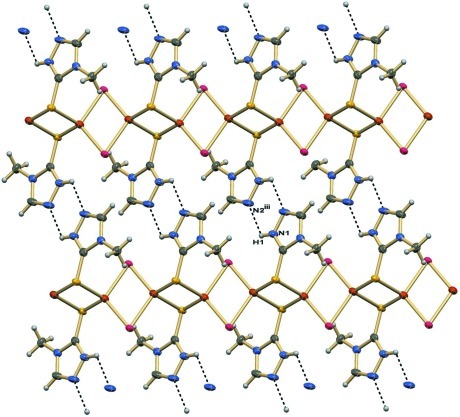
The inter-molecular hydrogen bonds generating 2-D sheet of [Cu(µ2-Hmptrz)(µ2-Br)]n.

## supplementary crystallographic information

### Comment

1,2,4-Triazole and its derivatives are very interesting ligands because they combine the coordination geometry of both pyrazole and imidazole with regard to the arrangement of their three heteroatoms. The interest in unsubstituted and substituted 1,2,4-triazole derivatives arise from their ability to bond metal ions in a various forms. A large number of mononuclear, oligonuclear and polynuclear metal coordination compounds with 1,2,4-triazole derivatives as ligands including the coordination chemistry have been described (Cingi *et al.*, 1996; Haasnoot, 2000; Kajdan *et al.*, 2000; Menzies & Squattrito, 2001; Klingele & Brooker, 2003).

Herein, we report the crystal structure of the title compound. The polymeric complex of [Cu(*µ_2_* –Hmptrz)(*µ_2_*-Br)]_n_ is isomorphous with those complex that has been report [Cu(*µ_2_*-Hmptrz)(*µ_2_*-I)]_n_ (Wang *et al.*, 2011). The chemical structure of this complex is shown in Scheme 1. Each Cu atom is a distorted tetrahedral geometry with the angles around Cu centre atom ranging from 104.74 (3)° to 117.72 (3)° and it is coordinated by two *µ*_2_-S donating Hmptrz molecules and two *µ*_2_-Br atoms. The one-dimensional chain built from two type of Cu(*µ*_2_-S)_2_ and Cu(*µ*_2_-Br)_2_ unit sharing the Cu centre atoms. Each pair of *µ*_2_-S and of *µ*_2_-Br bridges alternate to link between two Cu centre atoms giving the linked rhomboid of Cu_2_S_2_ and Cu_2_Br_2_ core forming a 1-D chain running along *a*-axis. Each Cu_2_S_2_ rhomboid is located at nearly perpendicular position to adjacent Cu_2_Br_2_ rhomboid with a dihedral angle of 86.90 (4)*^o^* between these planes. A view of the one-dimensional polymeric chain is shown in Figure 1.

The Cu···Cu distances of of Cu(*µ*_2_-S)_2_ and Cu(*µ*_2_-Br)_2_ unit are 2.8246 (9) and 2.7740 (9) Å. The latter distance is slightly shorter than the sum of van der Waals radii of Cu atoms (2.80 Å). The inter-molecular hydrogen bonds N(1)—H(1)···N(2)^iii^ [N(1)···N(2)^iii^ = 2.890 (4) Å, iii: -*x* + 1, -*y* + 2, -*z* + 2] between the adjacent 1-D polymeric chains are observed generating the two-dimensional sheets of supramolecular interactions running in *ac-*plane. The arrangement of the polymeric chains and the inter-molecular hydrogen bonds in crystal packing of this complex are shown in Figures 2 and 3, respectively.

### Experimental

The mixture of Hmptrz ligand (0.28 g, 2.43 mmol) and copper (I) iodide (0.15 g, 1.05 mmol) in acetronitrile solution was refluxed N_2_ gas. The yellow filtrate was allowed to stand at room temperature for 2 days. The block colorless crystals of [Cu(*µ_2_*-Hmptrz)(*µ_2_*-Br)]_n_ were isolated. This complex melts and decomposes at 234–235 *^o^*C.

### Refinement

All hydrogen atoms on carbon atoms were constrained, C—H = 0.95 Å with *U*_iso_(H) = 1.2*U*_eq_(C) for C-*sp*^2^ atoms of pyridine and phenyl rings and C—H = 0.98 Å with *U*_iso_(H) = 1.5*U*_eq_(C) for C-*sp*^3^ atoms of the methyl group, respectively. The hydrogen atom on N atom is located in a difference Fourier map and restrained, N—H = 0.86 Å with *U*_iso_(H) = 1.2*U*_eq_(N).

### Crystal data

**Table:**

[CuBr(C_3_H_5_N_3_S)]	*F*(000) = 496
*M**_r_* = 258.62	*D*_x_ = 2.450 Mg m^−^^3^
Monoclinic, *P*2_1_/*n*	Mo *K*α radiation, λ = 0.71074 Å
Hall symbol: -P 2yn	Cell parameters from 2999 reflections
*a* = 5.5781 (11) Å	θ = 2.6–27.5°
*b* = 12.931 (3) Å	µ = 9.02 mm^−^^1^
*c* = 9.810 (2) Å	*T* = 100 K
β = 97.69 (3)°	Prism, colorless
*V* = 701.2 (2) Å^3^	0.28 × 0.12 × 0.06 mm
*Z* = 4	

### Data collection

**Table:**

Bruker D8 CCD diffractometer	1610 independent reflections
Radiation source: sealed X-ray tube	1513 reflections with *I* > 2σ(*I*)
Graphite monochromator	*R*_int_ = 0.043
Detector resolution: 8.366 pixels mm^-1^	θ_max_ = 27.5°, θ_min_ = 2.6°
ω scans	*h* = −7→7
Absorption correction: multi-scan (*SADABS*; Bruker, 1998)	*k* = −16→16
*T*_min_ = 0.284, *T*_max_ = 0.582	*l* = −12→12
7830 measured reflections	

### Refinement

**Table:**

Refinement on *F*^2^	Primary atom site location: structure-invariant direct methods
Least-squares matrix: full	Secondary atom site location: difference Fourier map
*R*[*F*^2^ > 2σ(*F*^2^)] = 0.027	Hydrogen site location: inferred from neighbouring sites
*wR*(*F*^2^) = 0.056	H atoms treated by a mixture of independent and constrained refinement
*S* = 1.16	*w* = 1/[σ^2^(*F*_o_^2^) + (0.0145*P*)^2^ + 1.2302*P*] where *P* = (*F*_o_^2^ + 2*F*_c_^2^)/3
1610 reflections	(Δ/σ)_max_ = 0.001
86 parameters	Δρ_max_ = 0.64 e Å^−^^3^
1 restraint	Δρ_min_ = −0.37 e Å^−^^3^

### Special details

**Table:**

Geometry. All e.s.d.'s (except the e.s.d. in the dihedral angle between two l.s. planes) are estimated using the full covariance matrix. The cell e.s.d.'s are taken into account individually in the estimation of e.s.d.'s in distances, angles and torsion angles; correlations between e.s.d.'s in cell parameters are only used when they are defined by crystal symmetry. An approximate (isotropic) treatment of cell e.s.d.'s is used for estimating e.s.d.'s involving l.s. planes.
Refinement. Refinement of *F*^2^ against all reflections. The weighted *R*-factor *wR* and goodness of fit *S* are based on *F*^2^, conventional *R*-factors *R* are based on *F*, with *F* set to zero for negative *F*^2^. The threshold expression of *F*^2^ > 2*σ* (*F*^2^) is used only for calculating *R*-factors(gt) *etc*. and is not relevant to the choice of reflections for refinement. *R*-factors based on *F*^2^ are statistically about twice as large as those based on *F*, and *R*- factors based on all data will be even larger.

### Fractional atomic coordinates and isotropic or equivalent isotropic displacement parameters (Å^2^)

**Table:**

	*x*	*y*	*z*	*U*_iso_*/*U*_eq_	
Cu1	0.25060 (7)	0.99881 (3)	0.51220 (4)	0.01363 (11)	
Br1	0.02520 (5)	1.07438 (2)	0.68746 (3)	0.01177 (9)	
S1	0.52061 (14)	0.86702 (6)	0.58000 (7)	0.01143 (16)	
N1	0.5464 (5)	0.9389 (2)	0.8453 (3)	0.0139 (5)	
H1	0.417 (5)	0.976 (2)	0.838 (4)	0.017*	
N2	0.6910 (5)	0.9341 (2)	0.9706 (3)	0.0164 (6)	
N3	0.8341 (4)	0.83717 (19)	0.8149 (3)	0.0113 (5)	
C1	0.6309 (5)	0.8819 (2)	0.7499 (3)	0.0115 (6)	
C2	0.8636 (6)	0.8719 (2)	0.9480 (3)	0.0150 (6)	
H2	0.9944	0.8528	1.0154	0.018*	
C3	0.9938 (6)	0.7674 (2)	0.7517 (3)	0.0154 (6)	
H3A	0.9086	0.7025	0.7263	0.023*	
H3B	1.1385	0.7530	0.8173	0.023*	
H3C	1.0414	0.7999	0.6692	0.023*	

### Atomic displacement parameters (Å^2^)

**Table:**

	*U*^11^	*U*^22^	*U*^33^	*U*^12^	*U*^13^	*U*^23^
Cu1	0.01225 (19)	0.0179 (2)	0.01017 (19)	0.00017 (15)	−0.00072 (14)	−0.00002 (15)
Br1	0.01057 (15)	0.01533 (16)	0.00898 (15)	−0.00146 (11)	−0.00027 (10)	−0.00234 (11)
S1	0.0127 (4)	0.0118 (4)	0.0094 (3)	−0.0004 (3)	0.0001 (3)	−0.0016 (3)
N1	0.0140 (13)	0.0179 (14)	0.0093 (12)	0.0056 (11)	−0.0001 (10)	0.0004 (10)
N2	0.0215 (14)	0.0184 (14)	0.0081 (12)	0.0047 (12)	−0.0024 (10)	0.0004 (11)
N3	0.0116 (12)	0.0102 (12)	0.0118 (12)	0.0013 (10)	0.0005 (10)	0.0013 (10)
C1	0.0116 (14)	0.0094 (14)	0.0134 (15)	−0.0018 (11)	0.0008 (11)	0.0017 (11)
C2	0.0167 (15)	0.0165 (16)	0.0107 (14)	0.0034 (13)	−0.0020 (12)	0.0005 (12)
C3	0.0132 (15)	0.0172 (16)	0.0163 (16)	0.0052 (12)	0.0042 (12)	0.0005 (13)

### Geometric parameters (Å, º)

**Table:**

Cu1—S1	2.3124 (9)	N1—N2	1.378 (4)
Cu1—S1^i^	2.4012 (9)	N1—H1	0.862 (18)
Cu1—Br1	2.4638 (7)	N2—C2	1.296 (4)
Cu1—Br1^ii^	2.5085 (8)	N3—C1	1.354 (4)
Cu1—Cu1^ii^	2.7740 (9)	N3—C2	1.370 (4)
Cu1—Cu1^i^	2.8246 (9)	N3—C3	1.463 (4)
Br1—Cu1^ii^	2.5085 (8)	C2—H2	0.9500
S1—C1	1.708 (3)	C3—H3A	0.9800
S1—Cu1^i^	2.4012 (9)	C3—H3B	0.9800
N1—C1	1.327 (4)	C3—H3C	0.9800
			
S1—Cu1—S1^i^	106.40 (3)	C2—N3—C3	127.2 (3)
S1—Cu1—Br1	117.72 (3)	N1—C1—N3	105.0 (3)
S1^i^—Cu1—Br1	108.84 (3)	N1—C1—S1	129.5 (2)
S1—Cu1—Br1^ii^	104.74 (3)	N3—C1—S1	125.5 (2)
S1^i^—Cu1—Br1^ii^	106.25 (3)	N2—C2—N3	111.7 (3)
Br1—Cu1—Br1^ii^	112.19 (2)	N2—C2—H2	124.1
Cu1—Br1—Cu1^ii^	67.81 (2)	N3—C2—H2	124.1
Cu1—S1—Cu1^i^	73.60 (3)	N3—C3—H3A	109.5
C1—N1—N2	112.6 (3)	N3—C3—H3B	109.5
C1—N1—H1	129 (3)	H3A—C3—H3B	109.5
N2—N1—H1	119 (3)	N3—C3—H3C	109.5
C2—N2—N1	103.6 (3)	H3A—C3—H3C	109.5
C1—N3—C2	107.1 (3)	H3B—C3—H3C	109.5
C1—N3—C3	125.7 (3)		
			
S1—Cu1—Br1—Cu1^ii^	−121.64 (4)	N2—N1—C1—N3	1.1 (3)
S1^i^—Cu1—Br1—Cu1^ii^	117.29 (3)	N2—N1—C1—S1	−177.6 (2)
Br1^ii^—Cu1—Br1—Cu1^ii^	0.0	C2—N3—C1—N1	−1.1 (3)
Cu1^i^—Cu1—Br1—Cu1^ii^	172.54 (4)	C3—N3—C1—N1	−179.4 (3)
S1^i^—Cu1—S1—C1	93.28 (12)	C2—N3—C1—S1	177.7 (2)
Br1—Cu1—S1—C1	−29.05 (12)	C3—N3—C1—S1	−0.6 (4)
Br1^ii^—Cu1—S1—C1	−154.45 (11)	Cu1—S1—C1—N1	16.7 (3)
Cu1^ii^—Cu1—S1—C1	−97.63 (12)	Cu1^i^—S1—C1—N1	92.2 (3)
Cu1^i^—Cu1—S1—C1	93.28 (12)	Cu1—S1—C1—N3	−161.8 (2)
S1^i^—Cu1—S1—Cu1^i^	0.0	Cu1^i^—S1—C1—N3	−86.3 (3)
Br1—Cu1—S1—Cu1^i^	−122.33 (3)	N1—N2—C2—N3	−0.2 (4)
Br1^ii^—Cu1—S1—Cu1^i^	112.27 (3)	C1—N3—C2—N2	0.8 (4)
Cu1^ii^—Cu1—S1—Cu1^i^	169.09 (4)	C3—N3—C2—N2	179.1 (3)
C1—N1—N2—C2	−0.5 (4)		

Symmetry codes: (i) −*x*+1, −*y*+2, −*z*+1; (ii) −*x*, −*y*+2, −*z*+1.

### Hydrogen-bond geometry (Å, º)

**Table:**

*D*—H···*A*	*D*—H	H···*A*	*D*···*A*	*D*—H···*A*
N1—H1···N2^iii^	0.86 (2)	2.35 (3)	2.890 (4)	121 (3)
N1—H1···Br1	0.86 (2)	2.78 (2)	3.566 (3)	153 (3)

Symmetry code: (iii) −*x*+1, −*y*+2, −*z*+2.

## References

[bb1] Bruker (2003). *SMART*, *SAINT* and *SADABS* Bruker AXS Inc., Madison, Wisconsin,USA.

[bb2] Cingi, M. B., Bigoli, F., Lanfranchi, M., Leporati, E., Pellinghelli, M. A. & Foglia, C. (1996). *Inorg. Chem.* **95**, 37–43.

[bb3] Haasnoot, J. G. (2000). *Coord. Chem. Rev.* **200–202**, 131–185.

[bb4] Kajdan, T. W., Squattrito, P. J. & Dubey, S. N. (2000). *Inorg. Chim. Acta*, **300–302**, 1082–1089.

[bb5] Klingele, M. H. & Brooker, S. (2003). *Coord. Chem. Rev.* **241**, 119–132.

[bb6] Macrae, C. F., Bruno, I. J., Chisholm, J. A., Edgington, P. R., McCabe, P., Pidcock, E., Rodriguez-Monge, L., Taylor, R., van de Streek, J. & Wood, P. A. (2008). *J. Appl. Cryst.* **41**, 466–470.

[bb7] Menzies, C. M. & Squattrito, P. J. (2001). *Inorg. Chim. Acta*, **314**, 194–200.

[bb8] Sheldrick, G. M. (2008). *Acta Cryst.* A**64**, 112–122. 10.1107/S0108767307043930 18156677

[bb9] Westrip, S. P. (2010). *J. Appl. Cryst.* **43**, 920–925.

